# Context-guided segmentation for histopathologic cancer segmentation

**DOI:** 10.1038/s41598-025-86428-7

**Published:** 2025-02-13

**Authors:** Jeremy Juybari, Josh Hamilton, Chaofan Chen, Andre Khalil, Yifeng Zhu

**Affiliations:** 1 CompuMAINE Lab, Department of Chemical and Biomedical Engineering, University of Maine, Orono, 04469 USA; 2 DEAL Lab, Department of Electrical and Computer Engineering, University of Maine, Orono, 04469 USA; 3 School of Computing and Information Science, University of Maine, Orono, 04469 USA

**Keywords:** Pathology, Preclinical research

## Abstract

Microscopic inspection of histologically stained tissue is considered as the gold standard for cancer diagnosis. This research is inspired by the practices of pathologists who analyze diagnostic samples by zooming in and out. We propose a dual-encoder model that simultaneously evaluates two views of the tissue at different levels of magnification. The lower magnification view provides contextual information for a target area, while the higher magnification view provides detailed information. The model consists of two encoder branches that consider both detail and context resolutions of the target area concurrently for binary pixel-wise segmentation. We introduce a unique weight initialization for the cross-attention between the context and detail feature tensors, allowing the model to incorporate contextual information. Our design is evaluated using the Camelyon16 dataset of sentinel lymph node tissue and cancer. The results demonstrate the benefit of including context regions when segmenting for cancer, with an improvement in AUC ranging from 0.31 to 0.92% and an improvement in cancer Dice score ranging from 4.09% to 6.81% compared to single detailed input models.

## Introduction

Breast cancer is the second leading cause of cancer-related deaths in women, affecting one in every eight women over their lifetime^1,2^. Currently, the standard method for confirming a radiologist’s findings in mammography is through a biopsy, where pathologists visually examine stained tissue slides under a microscope^3^. The rapid evolution of digital imaging technology, specifically whole-slide imaging or virtual microscopy, has opened up new possibilities for the development of an AI-powered diagnostic assistant tool. This is particularly important, considering that two-thirds of the world’s pathologists are concentrated in only 10 countries^4^. Integrating an AI-powered assistant tool has the potential to greatly assist pathologists and improve cancer diagnosis, especially in countries where delays in diagnostics contribute to a significant number of cancer-related deaths (approximately 70% in India) caused by treatable risk factors^5^.

**Fig. 1:**
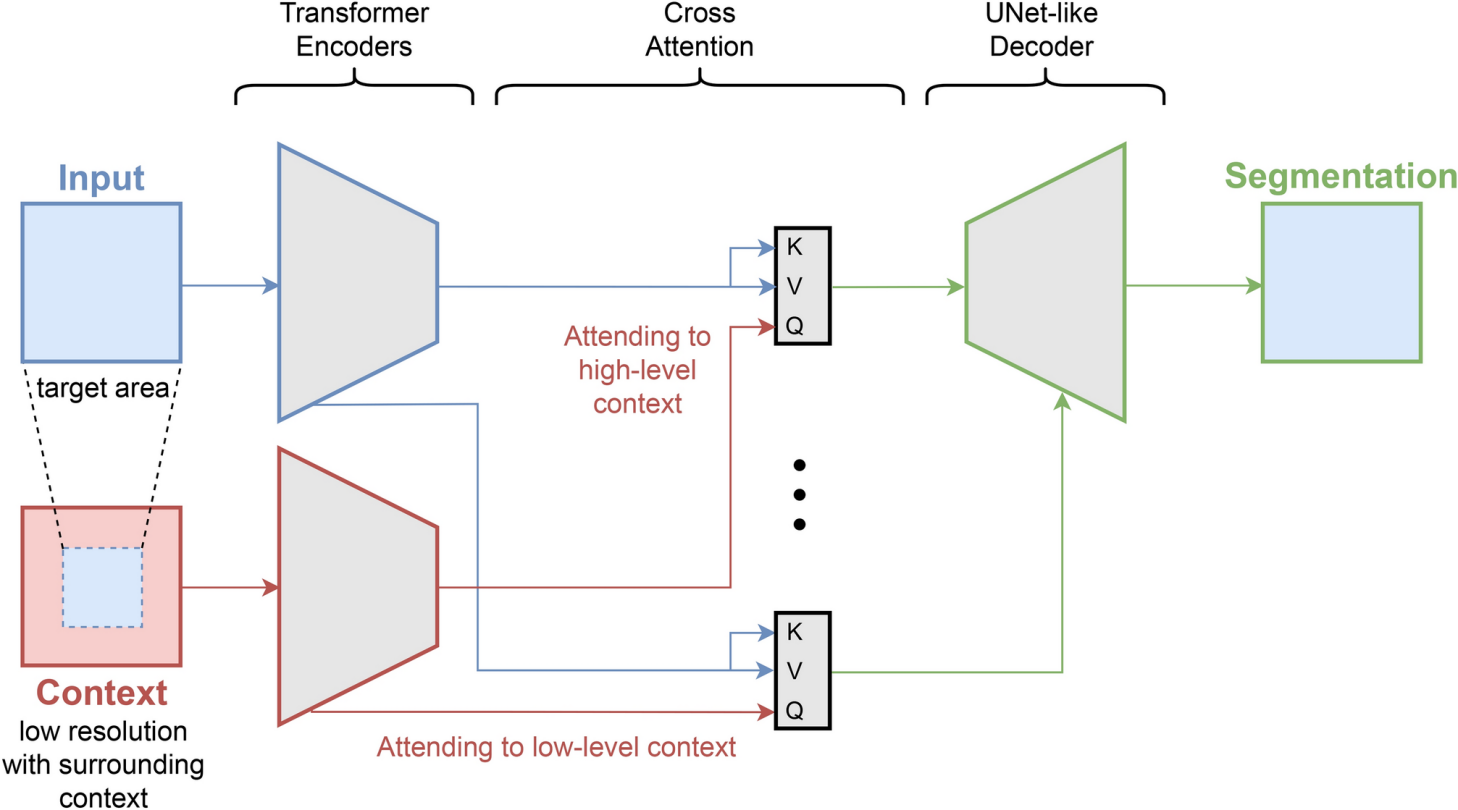
Overall architecture of our CGS-Net model. It takes two inputs: a higher-resolution patch of the target area and a lower-resolution context patch with a larger field of view. It consists of two transformer-based encoders and a UNet-like decoder. At each level, the cross-attention module incorporates the corresponding context information provided by the context patch encoder. In the cross-attention module, *K* (key) and *V* (value) are derived from the target area encoder, while *Q* (Query) is obtained from the context encoder.

To assist the diagnosis of breast cancer, this paper introduces a novel deep learning architecture called the *Context Guided Segmentation Network*, referred to as CGS-Net. Figure 1 shows the overall architecture of the model. The novelty of CGS-Net lies in that it is an end-to-end system that can automatically learn to incorporate context information for more accurate segmentation in medical images.

The architecture of CGS-Net is inspired by the sequential examination process employed by pathologists when analyzing tissue samples. This process involves two stages: an initial exploration at low magnification, followed by a detailed analysis at higher magnifications. This approach mimics the behavior of pathologists zooming in and out during slide navigation. During microscopic diagnosis, pathologists begin by observing the tissue at a low-resolution view to gather information, such as the spatial arrangement of the tissue, and search for abnormal areas. Once a suspicious area is identified, they increase the magnification to scrutinize the target region in finer detail. After thoroughly examining the area of interest, pathologists then zoom out to continue their examination of the remaining tissue, adjusting the magnification level as necessary.

The use of multi-resolution views in pathologist workflows has been a recurring theme in recent literature, made possible by advancements in computational power. As a result, complex multi-image and multi-encoder model architectures have been developed. The body of work in this area can be divided into two categories: binary semantic segmentation^6–10^, which focuses on differentiating between cancer and non-cancer tissue, and multi-class semantic segmentation^11–16^, where pixels are classified into specific types of tissue on a histopathological slide. These studies describe various networks that incorporate multi-resolution views in customized CNNs to achieve accurate pixel classifications. In each case, these networks employ two inputs with identical dimensions and center pixels but different resolutions. They also share structural similarities, such as the utilization of two or three encoder branches. Some studies in this area employ unique approaches to incorporate multi-resolution inputs, such as using the entire Whole Slide Image (WSI) as an input^8,14^, or applying adaptive weighting of the multi-input resolutions^15^. Less computationally intensive methods involve patch-level classification with a multi-resolution voting system^17^ or using multiple instance learning^18^. Another research direction is to combine a convolutional neural network with reasoning for an explainable AI approach while training on sparsely labeled samples instead of patches from the entire WSI^19^.

A more recent strategy in the literature is to use only lower-resolution views^9^, or to incorporate lower-resolution information from one input image using more advanced architectures, such as attention mechanisms^10^ or transformer models with CNN dual encoder architectures^16^. To the best of our knowledge, no existing literature combines the idea of lower-resolution attention and multi-input vision transformer architectures. Using a multi-resolution dual-encoder approach with cross-attention has the potential to outperform baseline models.

Another commonality observed in the literature is the absence of a publicly available, pre-organized, standard WSI histopathological dataset. This limitation hampers our ability to compare models effectively. The intensity of H&E staining is not consistent and there is a significant variation in staining appearance across different institutions, laboratories, and even within institutions^20^. Therefore, it is necessary to apply color transformation or augmentation strategies before comparing staining across different datasets. Another challenge lies in the need to extract smaller patches from the WSI. For example, the Camelyon16 dataset is commonly used for semantic segmentation of breast cancer^21^. Each patient slide in this dataset consists of gigapixel-size images, which need to be divided into smaller patches for model input. However, there is a lack of sufficient description of the patch algorithm in most papers we reviewed, hindering reproducibility. The following papers include enough information for their patch algorithm^7,9,12,16^.

The absence of these details makes it difficult to replicate experiments or compare different models and underlines the reliability of the results. Important factors, such as variations in microns per pixel due to different microscope scanning objectives used by whole slide scanners, are not accounted for. This motivates our work to design reliable and reproducible patch-extracting algorithms. We use these patches to test the performance of a transformer dual-encoder model using multi-resolution views with a unique cross-attention initialization designed in this paper.

Our focus is on developing a neural network architecture that closely replicates the pattern followed by pathologists during tissue sample analysis: utilizing low-resolution views for an initial overview and high-resolution views to revisit selected areas. This diagnostic pattern proves to be highly effective when dealing with input images that are thousands of times larger than the typical view field of a computer screen or a microscope. A lower-resolution view is instrumental in providing a broader field of view and additional context for identifying architectural features, structural texture, and spatial organizations. In contrast, a higher-resolution view facilitates a more detailed and accurate view of specific regions of interest. The amalgamation of multiple views with distinct resolutions is essential for enabling the human visual system to accurately identify cancer regions. Our CGS-Net architecture is designed to simultaneously incorporate multiple views with varying resolutions as inputs, synergizing the insights obtained from each view to facilitate more precise analysis.

Specifically, this work makes the following specific contributions:

(i) We demonstrate the effectiveness of incorporating tissue context into model detail predictions by using an additional encoder branch.
(ii) To the best of our knowledge, we are the first to introduce a novel training scheme that initially trains the detail and context encoders separately. After being trained separately, the encoders are then combined into CGS-Net for joint training.
(iii) We provide a unique weight initialization for the cross-attention mechanism, allowing CGS-Net to determine how to incorporate the context encoder branch.

## Results

### Dataset

**Fig. 2:**
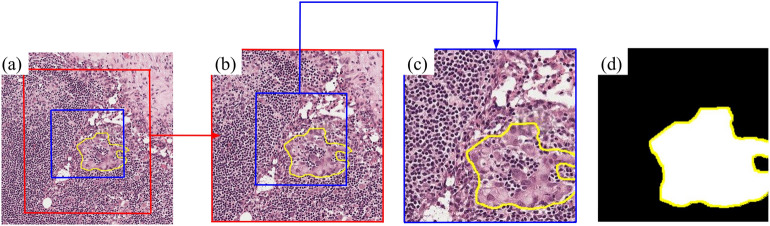
(**a**) Showing the different patch levels from a slide map. The yellow polygon separates the cancer and non-cancer tissue. The central red square represents the level-3 patch that was extracted from this slide, also shown in (**b**). The central blue square is the level-2 patch, also shown in (**b**) and (**c**). The corresponding level-2 patch mask is given in (**d**).

The dataset used in this study consisted of 153 cancer slides and 230 non-cancer slides which are H&E stained WSIs. The tumor annotations were performed by two patholoists on the WSIs. The Camelyon16 dataset had WSIs from two different scanners with different spatial resolutions. For more detail on the WSI scanners, please see the original Camelyon16 paper^21^. For each scanner, every slide is stored as a TIFF file and contains multiple magnifications, referred to as levels. For example, from one scanner: full resolution (level-0, 0.243 μm/pixel ), reduced magnification by a factor of 4 (level-1, 0.972 μm/pixel), by 16 (level-2, 3.89 μm/pixel), by 32 (level-3, 7.8 μm/pixel), and so on. Going between levels is similar to a pathologist halving their objective magnification, for example going from a 20x objective to a 10x objective. To address the memory requirement challenge, a common approach is to extract patches, which are smaller subregions of the WSI^6,22^. In this study, all patches were standardized to a size of 224 × 224 × 3 pixels (RGB images). The patch collection strategy is explained in detail in Methods. For a visual depiction of the distinction between level-2 and level-3 patches, please refer to Fig. 2.

Figure 2a showcases a small subset of a slide image, with a yellow polygon separating the cancer and non-cancer tissue. The central red box in Figure 2a represents a level-3 patch. Inside this red box, there is a smaller blue box, which corresponds to the level-2 patch. Both the level-3 and level-2 patches have dimensions of 224 $\times$ 224 pixels and share the same center pixel. Figure 2b displays only the level-3 patch (red) with the level-2 patch (blue) contained within it. Figure 2c shows the level-2 patch, illustrating the difference in detail between the level-3 and level-2 patches. In the level-2 patch, the cell nuclei are distinctly visible (roughly circular dark purple areas), whereas the level-3 patch encompasses the level-2 patch and its surrounding tissue at a lower resolution. Figure 2d represents the corresponding level-2 patch mask in (c), where white indicates cancer regions and black represents non-cancer regions. For a breakdown of cancer and non-cancer patches into training, validation, and testing sets, refer to Supplementary Table 1.

### CGS-Net outperforms single encoder

Mixed Transformer encoders (MiT) and Swin V2 encoders were implemented as the transformer encoders for CGS-Net as shown in Fig. 1. To control for the number of parameters in the model, a small and large version of each encoder was used. Such a setup allows for a comparison of single detail inputs models and CGS-Net as a function of encoder type and size. The Area Under the Curve (AUC) statistic was used to evaluate the different models. Recall that AUC is independent of probability thresholding, which makes it robust to compare across models; however, it is not as sensitive to Type I Error^23^. The Dice score is much more sensitive to such errors^23^ and is included using a threshold of 0.5 across all models allowing for easy comparison across models. The difficult cancer class was oversampled with overlapping patches as described in Extracting Patches in Methods. Each pixel and binary label from all patches were used to calculate the receiver operating characteristic curve (ROC) and the cancer Dice score. This means that some pixels in cancer patches have multiple occurrences in the metrics. The non-cancer patches are not oversampled, however, a non-cancer pixel in a cancer patch may also have multiple counts.

Table 1 shows the best model combinations, for the single-input and dual-input models. Please see Supplementary Fig. 3 for the full ROC curve plot. CGS-Net models had a higher AUC and cancer class Dice score for a similar hyperparameter set and across encoder size and type. The best instance of CGS-Net was with the MiT-B1 encoders which yielded an AUC of 98.1% and a cancer Dice score of 70%, outperforming every other model including other CGS-Net models. It outperformed a single detail model using the same encoder by 0.47% and the larger encoder of the same type by 0.88% in AUC scores. The cancer Dice scores followed a similar pattern with a 4.09% improvement over the baseline and 12.85% increase over an encoder of a larger size. A drawback of the Dice score is the need to optimize the threshold for predictions. This leads to the higher standard deviations as shown in Table 1. The AUC is independent of thresholds, providing more consistent results and thus smaller standard deviations. The CGS-Net models consistently outperformed their single-input baselines across model architectures and sizes. Better performance may exist for a different combination of techniques preventing overfitting. The Swin V2 encoders do not have any smaller sizes, so the performance gap between the MiT and Swin V2 encoders may result from the parameter count and not the architecture.

**Fig. 3:**
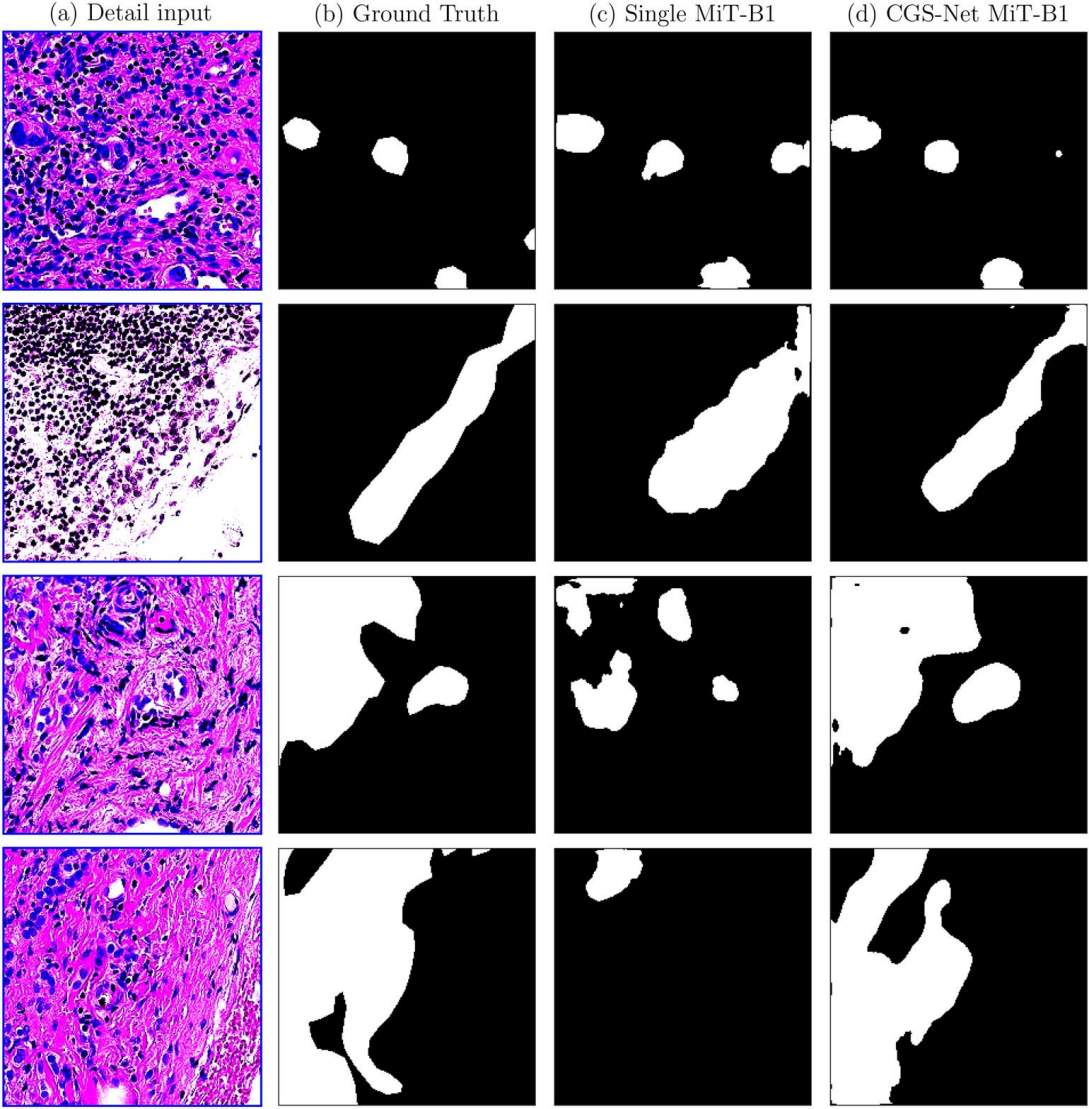
(**a**) An example of an input detail patch. (**b**) The ground truth mask for the input patch with black representing non-cancer tissue and white representing cancer tissue. All these patches contain both cancer (white) and non-cancer (black). The segmentation maps are predicted by a single-input network (**c**) and by the dual-input network, CGS-Net (**d**).

Figure 3 shows some example outputs across the best single-input and CGS-Net models (MiT-B1). The first row shows an example where the CGS-Net outperformed the benchmark. The false positives were smaller, however, both models missed some cancer. The remaining three rows qualitatively show the efficacy of the CGS-Net architecture.

**Table 1:** Performance comparison of CGS-Net models vs. baseline models for cancer segmentation. The CGS-Net models outperformed their single-input baseline models. These results were seen across AUC and cancer Dice scores for all model sizes and architectures.

Model	Parameters	AUC	CGS-Net Improvement	Cancer Dice score	CGS-Net Improvement
Single MiT-B1	15.14 M	$0.976 \pm 0.0008$	-	$0.6677 \pm 0.108$	-
CGS-Net MiT-B1	30.08 M	$\mathbf {0.9806} \pm \mathbf {0.0002}$	0.47%	$\mathbf {0.7004} \pm \mathbf {0.1228}$	4.09%
Single MiT-B2	26.18 M	$0.972 \pm 0.002$	-	$0.5719 \pm 0.0326$	-
CGS-Net MiT-B2	52.17 M	$\mathbf {0.979} \pm \mathbf {0.0006}$	0.72%	$\mathbf {0.6036} \pm \mathbf {0.0098}$	5.54%
Single SwinV2 Tiny	29.56 M	$0.971 \pm 0.0009$	-	$0.5735 \pm 0.04$	-
CGS-Net SwinV2 Tiny	60.81 M	$\mathbf {0.974} \pm \mathbf {0.0015}$	0.31%	$\mathbf {0.6126} \pm \mathbf {0.0035}$	6.81%
Single SwinV2 Small	50.92 M	$0.967 \pm 0.0097$	-	$0.5631 \pm 0.029$	-
CGS-Net SwinV2 Small	103.54 M	$\mathbf {0.976} \pm \mathbf {0.0008}$	0.93%	$\mathbf {0.5936} \pm \mathbf {0.0066}$	5.42%

The bold denotes the best performing model within each pair.

### Multi-cross attention

It is important to note that the weights for the multi-cross attention modules (MCA) were initialized with a value of one for the detail feature tensors (keys and values) while the weights for the input context feature tensors were initialized with zero (queries). As a result, upon weight initialization, CGS-Net is similar to a single encoder-decoder model for the detail resolution. This allows the model to determine how to incorporate the context information. Table 2 presents the average weights for the queries, keys, and values for the CGS-Net MiT-B1 model. It is worth noting that the queries, keys, and values all contain weights of similar magnitude. Please see the supplement for the averages along with their standard deviations. Based on this, we can conclude that the model has learned to effectively incorporate the context.

**Table 2:** Weight analysis of multi-cross attention modules in the final CGS-Net weights. The queries were initialized to 0 while the keys and values were initialized to 1. The average value of the weights is provided.

Cross	Queries	Keys	Values
Weight init	0	1	1
First cross	3.43e−09	− 1.42e−08	2.59e−09
Second cross	7.88e−09	− 2.33e−09	9.13e−09
Third cross	− 4.84e−10	− 3.63e−09	− 2.97e−09
Fourth cross	1.85e−09	− 1.41e−09	− 8.12e−10

## Discussion

CGS-Net outperformed a single-input model of a similar size and learned how to integrate information from the context branch across two separate transformer encoder backbones. The baseline single-input models ignore the surroundings of the examined area. Our model, CGS-Net, successfully mimicked how a pathologist looks at histological samples, by using two encoders that simultaneously examine two views of different levels of magnification power. When inspecting a specific region, CGS-Net also considers its surrounding regions, which provide a wider cellular and histological context for the examined region. CGS-Net also demonstrates the importance of how to handle the training and combination of dual input models. The MCA was initialized with a zero for context inputs and one for detail inputs. After the entire training procedure, if the detail inputs were solely sufficient, one would expect their weights to be of differing magnitudes. However, as we demonstrate the model successfully learns to incorporate both the context and detail branches. This matches our hypothesis that mimicking pathologist workflows would improve model performance. Lastly, our unique approach to forcing CGS-Net to first train the MCA on two separately pre-trained encoder branches allows the cross-attention weights to determine the optimal combination of context and detail inputs. We believe multi-input neural networks should be designed so that models learn, through zero weight attention, how to incorporate the multiple inputs.

Our study was limited to only two image resolutions and two vision transform architectures. There is potential for further exploration by incorporating more resolutions in the network or even using the entire WSI^8,12,14^ and testing a wider range of architectures with our cross-attention strategy. Please see Supplementary Fig. 4 for an example of whole slide inference. Additionally, the Camelyon16 dataset only provides annotations for the cancer class^21^. However, there are various other types of tissues, such as stromal and necrotic tissues, which could be useful for pathologists and are present in other datasets^11–16^. This opens up another avenue for exploration by evaluating the performance of CGS-Net in multiclass tissue segmentation. Furthermore, there are histological images available for multiple cancer and non-cancer disease contexts allowing for exploration of transfer learning capabilities. A comprehensive model that accurately simulates a pathologist’s workflow should have strong transfer capabilities, as pathologists are trained in multiple disease contexts.

It is well-known that incorporating multimodal data, such as radiological and molecular profiling along with H&E stains^24,25^, can significantly improve model accuracy. We believe that our approach, which utilizes multi-resolution views with cross-attention, can be adapted for handling multimodal data.

## Methods

### Context-guided segmentation (CGS-Net) architecture

**Fig. 4:**
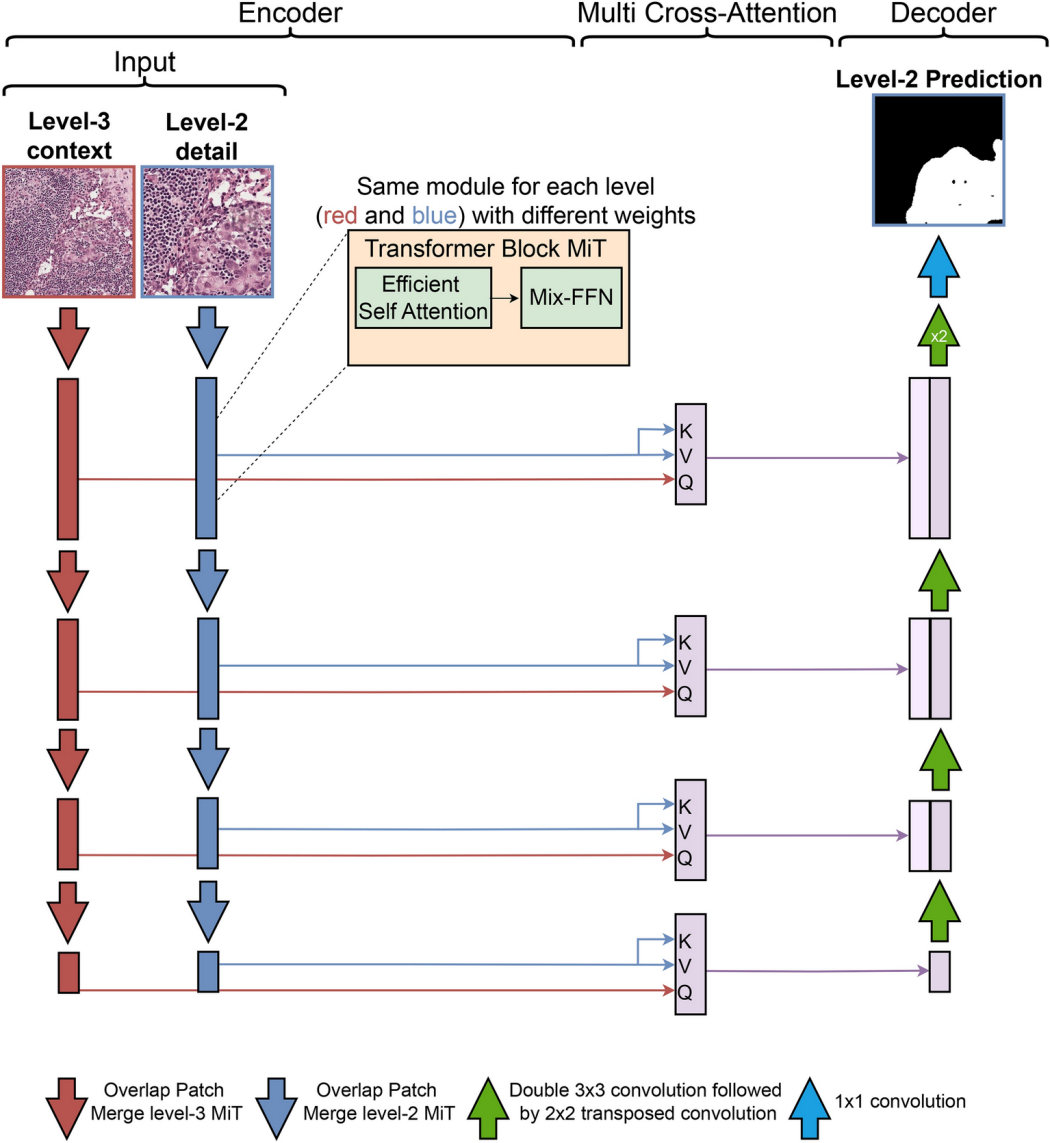
The architecture of CGS-Net for semantic segmentation using MiT-B1 encoders^28^, which takes into account both details and context. The two input images with the same dimensions are the level-2 high-magnification (represented by blue), providing a focused view, and the level-3 low-magnification (represented by red), providing a wider context. The encoder for the detail shown in blue and the encoder for the context shown in red. The center of the figure is the cross attention blocks, where the context feature tensors serve as the query input and the detail feature tensors are the keys and values input. The right side of the figure shows the decoder, which extends the standard U-Net decoder by adding two additional double convolutions and transposed convolutions.

CGS-Net draws inspiration from the U-Net^26^ and dense multi-path U-Net^27^. The CGS-Net can be used with different encoder neural networks. We explore two different transformer-based encoders with two different sizes: Mixed Transformer encoders (MiT)^28^ and Swin Transformer V2 encoders^29,30^. The decoder architecture is an extended U-Net style decoder. A visual representation of the CGS-Net architecture, which uses a MiT backbone, is shown in Fig. 4. However, the overall architecture remains approximately the same for different backbones.

The MiT encoders utilize overlapped Patch Merging, which differs from the patch embedding approach employed in Vision Transformers (ViT). This distinction enables the MiT encoder to perform down-sampling while maintaining hierarchical feature maps^28^. Moreover, the MiT encoders incorporate efficient self-attention mechanisms, which use smaller weight sizes for keys while preserving the same shape for input and output^28^.

The Swin V2 encoders are also implemented as a backbone. Ref.^30^ developed a shifting window strategy that constrains attention to non-overlapping local image windows (a collection of image patches) while enabling cross-window interactions. The original Swin Transformer modified the self-attention operation to include weights for positional information of the image windows^30^. Swin V2 utilizes residual post normalization and scaled cosine attention for scaling up the model capacity^30^. Additionally, the window resolution is increased by using continuous relative position bias and log-space coordinates^30^.

The decoder structure aligns with the U-Net decoder but includes two additional transposed convolution layers. These additional layers upsample the feature vector from the encoder back to the original input image shape. This decoder structure was used with both the MiT and SwinV2 backbones. Notably, unlike the approach in Ref.^28^, we abstain from down-sampling the ground truth for loss functions and metric calculations. This decision was deliberate, considering that cancer regions already constitute a very small proportion (1%) of the entire slide. Furthermore, down-sampling by a factor of 4 in each direction would lead to a reduction in the limited cancer class pixels.

In line with previous studies^6,11,12^,CGS-Net incorporates two parallel encoders, each taking one view as input. These views have the same size but differ in resolution, corresponding to the level-2 and level-3 patches as described in Methods. It is important to note that both patches share the same center pixel, with the level-3 patch having a lower pixel-resolution by a factor of 2 in both the *x* and *y* directions. The level-3 patch encompasses the level-2 patch along with the surrounding tissue at a lower resolution. Referring to the terminology used in Ref.^11^, the level-3 encoder path can be described as the context branch, while the level-2 encoder path is referred to as the target or detail branch. The level-2 patches contain detailed information about the cells, while the level-3 patches provide context by encompassing the surrounding tissue architecture.

In the CGS-Net architecture, each encoder path adopts the MiT-B1 model. However, it is worth noting that the design allows for the utilization of any encoder, provided that the feature vector shapes are appropriately adjusted to accommodate the varying embedding sizes. The central portion of Fig. 4 illustrates the Multi-Cross Attention (MCA) modules. In this configuration, the output from the detail encoder branch serves as both the values (*V*) and keys (*K*), while the output from the context encoder branch serves as the queries (*Q*) for the cross-attention modules.

This design adheres to the general principle that context information should guide the attention mechanism for the detail input. Notably, the weights applied to the MCA modules are initialized using a novel approach. Specifically, the query matrix (*Q*) is initialized with a zero matrix, while the identity matrix is applied to the keys (*K*), values (*V*), and the final projection layer. A detailed explanation of this initialization process can be found in the following section pertaining to the cross-attention module. This initialization strategy configures the MCA to initially focus solely on the input detail image, and the model subsequently learns how to effectively incorporate context information.

To the left of Fig. 4, the level-2 and level-3 input patches can be observed, each feeding into their respective color-coded encoder paths. Red corresponds to feature maps associated with level-3, while blue indicates the level-2 feature maps. The feature maps maintain their color coding as they enter the cross-attention module. The presence of light purple signifies that the feature maps from both encoders have undergone a cross-attention operation, reflecting their combined representation in the architecture. This fusion of information from different resolutions is a crucial aspect of the CGS-Net design.

### Cross attention for detail with context inputs

The concept of attention, as introduced in Ref.^31^, is used to correlate the relationship between inputs. Self-attention occurs when both inputs into the attention mechanism are the same, whereas attention or specifically cross-attention refers to the same attention mechanism applied to different inputs. One input (context) is multiplied with the *Q* weights while the other input is used for *K* and *V* respective keys and values weights (detail). In CGS-Net the Multi-Cross Attention (MCA) modules are specifically initialized with the weights for the context input zeroed and the weights for the detail input set to one. In other words, CGS-Net is initialized as a single encoder model. The rationale behind this initialization is to allow the context input to guide the detail input.

The weights for the cross attention are initialized as follows for a square input matrix $x_{n \times n}$

$$\begin{aligned} Q^{C}_{n\times n}= \,& 0_{n \times n}\\ K^{D}_{n\times n}= & V^{D}_{n\times n}= I_{n \times n} \end{aligned}$$

where $Q^{C}$ is the query weight matrix for the context input. For the detail input, we have $K^{D}$ and $V^{D}$ which are the weight matrices for the keys and values matrices for the detail input. The *C* and *D* designations are added for further distinction.

The cross-attention can be calculated as follows:

$$\text {Cross Attention}(Q^C,K^D,V^D) = \text {softmax}\left( \frac{Q^C(K^{D})^T}{\sqrt{d_k}}\right) V^D= \text {softmax}\left( 0_{n \times n}\right) V^D.$$

where $Q^C=0_{n \times n}$. Recall that the softmax is defined, for an input *z* as

$$\text {softmax}(z)= \frac{\exp (z)}{ \sum _j \exp (z_j)}$$

It follows that $\text {softmax}(0_{n \times n})$ becomes matrix *M* where each row sums to one with values 1/*n*. So now we have,

$$\text {softmax}(0_{n \times n})V=MV^D$$

The resulting matrix has the average value of the column, as every element in the column. Precisely,

$$V^D = \begin{bmatrix} v_{0,0} & \cdots & v_{0,n}\\ \vdots & \cdots & \vdots \\ v_{n,0} & \cdots & v_{n,n} \end{bmatrix} = [V^D_1 \cdots V^D_n]$$

where $V^D_i$ is the i-th column of $V^D$.

$$MV^D = \begin{bmatrix} \frac{1}{n} & \cdots & \frac{1}{n}\\ \vdots & \cdots & \vdots \\ \frac{1}{n} & \cdots & \frac{1}{n} \end{bmatrix} [V^D_1 \cdots V^D_n] = [\bar{V^D_1} \cdots \bar{V^D_n}].$$

where $\bar{V^D_{j}}$ is a column vector where every element is the average of $V^D_j$. The values (*V*) match the detail encoder initialization so CGS-Net training begins similarly to a single detail input model. The context input can not be the values *V*, since that would produce $\bar{V^C_{j}}$ which is not comparable to a single detail input model. The other requirement for initialization is inputs in the softmax function must equal zero. This means either the queries or keys must be set to zero.

### Slide dataset and splits

Whole slide imaging, which originated in the late 1990s, involves the digitalization of entire slides containing biopsy tissue samples. This process entails scanning a glass slide of stained tissue samples using a specialized scanner to create a very large digital image that covers the entire slide’s area^32^. Traditionally, pathologists inspect glass slides with tissue samples under a microscope. However, with ongoing advancements in digital scanning technology and visualization tools, WSI not only transforms clinical practices in medical diagnosis but also opens up promising avenues for the development of intelligent digital pathology utilizing modern AI^32^.

WSI poses two significant challenges. Firstly, each WSI image is exceptionally large, often exceeding 10 gigapixels. Secondly, WSIs may contain variations in color and artifacts introduced during the staining and image processing stages^32^. These artifacts can result in different types of tissue distortions, such as blurring and tissue separation^33^. Such artifact regions can be addressed through a quality control process, which we will describe in detail later.

The de-identified WSIs of sentinel lymph node tissues used in our study are sourced from the Camelyon16 challenge^21^. This challenge offers a publicly available dataset consisting of approximately 399 H&E stained whole slide images^21^. However, 16 of these slides were excluded primarily because their resolution levels did not match the other slides. These slides were independently obtained from two medical centers in the Netherlands. Each slide is stored as a TIFF file and contains multiple magnifications, referred to as levels: full resolution (level-0), magnification reduced by a factor of 4 (level-1), by 16 (level-2), by 64 (level-3), and so on. It’s important to note that the reduced magnification factors correspond to a reduction in area. Out of the 383 slides, 230 do not contain cancer, while 153 slides feature cancer regions identified by XML coordinates. No other tissue classes such as stromal, or necrotic are marked in this dataset. In Supplementary Fig. 1 provides visual representations of the pathologist’s demarcations. For each slide image, three distinct masks were generated: a cancer mask, a non-cancer mask, and a tissue mask, all displayed in Supplementary Fig. 1. These masks played a crucial role in patch quality control.

We divided the data into a 60–20–20 split for training, validation, and testing. The original Camelyon16^21^ dataset comprised 270 slides for training and 130 slides for testing, with the large test dataset serving as the basis for algorithm evaluation during the Camelyon16 Grand Challenge competition^21^. However, it is worth noting that this competition is no longer active, prompting us to revisit the data split. We sampled from three types of slides: non-cancer, cancer, and a category described as “cancer unique.” The latter category was created to differentiate between slides with small tumors and slides with larger tumors. A small tumor fits within a single patch whereas a large tumor requires multiple patches. The patch extraction process would produce more differentiated patches for larger tumors while smaller tumors would have patches with much more overlap, hence the term cancer unique. Without such a category, the distribution of patches in the dataset splits varies drastically, leading to results dependent upon the split rather than the underlying dataset. Random sampling was conducted for each category.

An important design decision was to perform the train-test data split at the slide level, rather than at the patch level. This approach was intentionally chosen to prevent potential information leakage from the training dataset into the test dataset. Partitioning datasets at the patch level could lead to different patches from the same slides being used for both training and testing, inadvertently exposing information about those slides during testing. This could result in the model learning incorrect signals and overestimating its performance.

### Extracting patches

The first step in patch extraction involved generating a tissue mask to prevent patches from predominantly containing background. This process was carried out by converting the RGB WSI to HSV and applying Otsu thresholding on the saturation mask. To ensure the inclusion of adipocytes, which were marked as non-tumor by pathologists, binary morphological closing was used with some mask clean-up steps (such as removal of small holes and objects).

There were two categories of WSIs: slides without cancer annotations known as non-cancer slides, and slides with cancer annotations known as cancer slides. Once the tissue masks were created, non-cancer and cancer masks were generated. This was achieved by using the cancer annotations as the cancer mask and considering any tissue mask area outside the cancer annotations as the non-cancer mask. It is important to note that the slides may contain fat tissues that can appear as holes in the tissue mask. Hence, these regions should not be excluded when creating slide-level masks as adipocytes can be present within cancerous and non-cancerous areas.

To create the final patch dataset, we extracted patches from each slide by proposing center pixels for level-2 patches, the higher resolution patches, and assessing the patch’s tissue composition by referencing the corresponding patch tissue masks (non-cancer, cancer, and tissue) extracted from the whole slide masks. This patch generation process varied depending on the patch type. For instance, non-cancer patches had random center pixels selected from the entire slide, while cancer boundary patches were sampled randomly along the XML boundary within a specific range of 112 pixels. There were multiple guidelines in place for which patches are kept and which category they belong to, and an overview of our strategy is shown in Supplementary Fig. 2. We classified five distinct types of patches: non-cancer patch from a non-cancer slide, non-cancer patch from a cancer slide, cancer patch, cancer boundary patch, and non-cancer island boundary patch. All patches had to contain more than 75% tissue as determined by the tissue mask in order to prevent sampling of the background unless the cancer annotations were along the glass boundary. Additionally, a critical constraint was applied: any two level-2 patches on the same slide could not overlap by 50% or more. This constraint was intended to distribute patches evenly and prevent unintentional redundant patches.

Specifically, a cancer patch is one where patches are sampled from the center pixels of cancer annotations. In the first pass, these patches contain at least 75% cancer tissue, and in the second pass, they must contain a minimum of 45% cancer tissue. In this group, it is possible for the entire patch to reside within a cancer region, *i.e.*, where the patch mask is entirely white. However, this necessitates a large cancer region, which was not present in most slides. There are two types of non-cancer patches: those from slides with no cancer and those from slides with cancer regions. Both types contain a minimum of 75% non-cancer tissue. Lastly, boundary patches feature patch masks that encompass both black and white regions, signifying the inclusion of both cancer and non-cancer tissue, as demonstrated in Fig. 2d. Two categories of boundary patches exist which are specifically extracted with the center pixel near a pathologist annotation demarcation to create more challenging classification problems for the model. These categories are cancer boundary patches, extracted from the edge of a pathologist’s cancer area annotation, and non-cancer island boundary patches, extracted from the edge of a pocket of normal tissue within a cancer area. The process for boundary patches involved three stages for patch generation. In the initial pass, a cancer tissue requirement of 40–75% was enforced. Following 10,000 iterations, this criterion was relaxed to a range of 20–75%, while the tissue requirement remained at 50%. During the final few thousand iterations, provided the required number of patches had not yet been collected, the criteria were further eased to 90 and 10% for cancer tissue and 25% for the tissue requirement.

To maintain a balanced contribution of patches from each slide, the number of patches generated per slide was determined according to the following guidelines: 50 non-cancer patches per cancer slide, 25 cancer patches per cancer slide, 50 cancer boundary patches, 25 non-cancer island boundary patches, and 25 non-cancer patches per non-cancer slide. Patch generation for a specific type was halted once either the maximum number of patches per slide was reached or after 25,000 center pixel coordinates were attempted for level-2 patches. If the minimum number of patches required for a specific type, such as cancer boundary patches, was not achieved, the remaining number of patches was added to the closest category, in this case non-cancer island boundary patches. The objective was to obtain approximately 100 patches containing cancer per cancer slide and to balance the amount of non-cancer from cancer slides with non-cancer from non-cancer slides. In order to ensure adequate coverage of slides with very small cancer regions, 10 pairs of XML cancer coordinates were randomly selected as patch centers for cancer boundary regions. Finally, in cases where fewer than 80 cancer patches were generated after the patch generation process, the XML coordinates were randomly sampled until 80 patches from that slide were made. These patches disregarded the tissue requirements, cancer tissue requirements, and the overlap constraints. This oversampling strategy for these regions was intentional, serving as a means of achieving class balance.

Level-3 patches were generated by simply down-sampling the center coordinates and extracting a patch with the down-sampled coordinates from the level-3 WSI resolution. In total, the dataset comprised 26,319 patches, distributed as 15,834 patches for training, 5180 for validation, and 5305 for testing. A detailed breakdown of these patches is provided in Supplemental Table 1.

### Novel training algorithm

All training was performed on an NVIDIA A100 SXM4 with 40 GB of memory. All models used Adam as an optimizer^34^. CGS-Net used four separate Adam optimizers: one for the encoder detail branch, encoder context branch, decoder, and the Multi-Cross Attention (MCA) modules. There were also three learning rate schedulers: one for encoders, decoder, and the MCA. The single-level models were trained first. A MiT-B1 with the extended decoder was trained using level-2 images and masks. A separate single model was trained using the level-3 data. The single-level models were initialized with ImageNet weights. The decoders followed the default weight initialization in PyTorch. The encoder was frozen for 600 epochs, and the entire run time was 1000 epochs for the single-level models. We then combined the training dataset with the validation dataset and retrained the model until the epoch where the lowest validation loss occurred. Finally, the test data were passed to the model to get the test statistics.

The weights from the encoders for both the single models (level-2 and level-3) were then transferred to the CGS-Net encoders. The decoder from the single level-2 model was also transferred since the ground truth in the CGS-Net model was the level-2 mask. The weights for the MCA are uniquely initialized as described in cross-attention in Methods. Thus, when CGS-Net started training the results were similar to the single level-2 model since weights had been transferred and MCA weight initialization set the context (level-3) inputs to zero. The CGS-Net training followed the same process as the single-level models, except that the encoders and the decoder were frozen for only 300 epochs. The frozen encoders and decoder allow the MCA weights to be trained on only combining the detail and context inputs. At 300 epochs the entire model was unfrozen and trained for 300 more epochs for a total of 600 epochs. The training and validation datasets were combined and the model was retrained on the combined dataset until the epoch that achieved the lowest validation loss. Then the test data were analyzed. The loss function was from Ref.^35^, which is a 95% weight on the focal loss and a 5% on the dice loss. All networks were developed with PyTorch^36^.

### Implementation details

The following regularization techniques were investigated to fight against overfitting: 2D dropout, L1 & L2 regularization, and data augmentation. The data augmentation was kept constant across the different models. Please see the supplement for specific implementation details.

The data augmentation included transpose, coarse dropout, Gaussian blur, rotation, horizontal flip, hue saturation value, vertical flip, affine transformations, and final normalization. We followed a hard color augmentation for training to mitigate the effects of staining variability across WSI slides^9^.

The values for L1 and L2 loss were determined empirically. Higher L1 and/or L2 weights lead to underfitting. Note that L1 and L2 were only applied to model components that did not contain pre-trained weights and were not frozen at the start of training. For instance, if L1 and L2 regularization were applied to the entire model but only the decoder was unfrozen then the decoder would have the weight penalty of the entire model weights and not just the decoder weights. We also applied L1 and L2 to only the decoder and then applied L1 and L2 to the encoder once it was unfrozen but this did not yield lower validation loss values. Instead, the validation loss spiked when the model unfroze, which logically follows from the L1 and L2 loss both increasing. However, we believe this disrupted the connection between the encoder and decoder since the encoder weights suddenly changed when unfrozen. Dropout was applied to the decoder and the MCA, and it was kept around 15%. The SwinV2 and MiT models had the same overall regularization strategy but with different hyperparameter values (see supplement).

## Supplementary Information

Supplementary Information.

## Data Availability

The train, validation, and test patch splits are publicly available for download from: https://web.eece.maine.edu/~zhu/CGS_Data/ The entire Camelyon16 dataset is publicly available: https://camelyon17.grand-challenge.org/Data/

## Code availability

The provided code includes everything needed to run the models and to generate the patch splits from the Camelyon16 dataset as was done in the paper. The code is available on Github: https://github.com/jeremyJJB/CGS-Net. The model weights are publicly available for download: https://web.eece.maine.edu/~zhu/CGS_Data/.
