# Supplementary material for: Context-guided segmentation for histopathologic cancer segmentation
Source: Sci Rep. 2025 Feb 13;15:5404. doi: 10.1038/s41598-025-86428-7 (PMC11825859; doi:10.1038/s41598-025-86428-7)
Supplement: Supplementary file 1 — Supplementary Information. [file 41598_2025_86428_MOESM1_ESM.pdf]

Supplementary Information

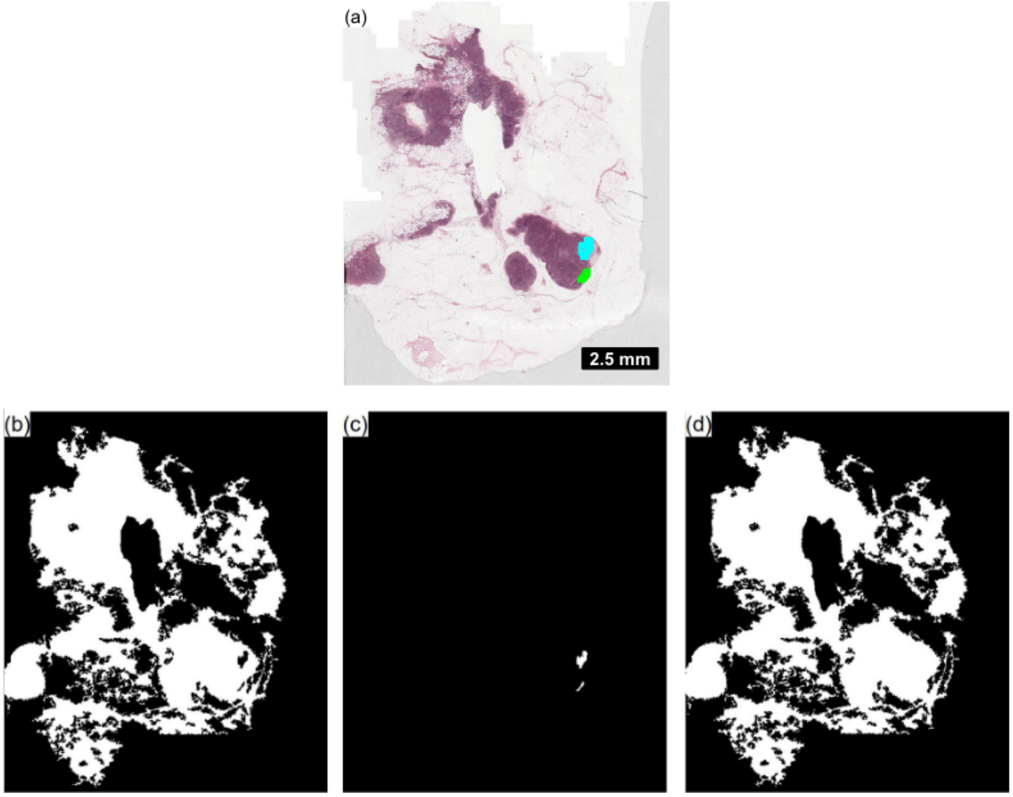

Supplementary Figure 1. (a) An example of a slide with a pathologist's annotation of the cancer boundary. The green and light blue markings represent the pathologist's annotations of metastases in this slide. The color is used to differentiate the cancer group markings. (b) A binary mask of the non-cancer tissue. (c) A binary mask of the cancer tissue. (d) A binary mask of the tissue that includes both cancer and non-cancer tissue. All the non-annotated regions are considered non-cancerous, and all the marked regions belong to the cancer class. This forms our cancer classification problem as a binary segmentation challenge. This example WSI had a size of $97,469 \times 220,452$ pixels at level-0, the full resolution (*i.e.,* 21 Gigapixels). At level 2, the slide dimensions are $24,367 \times 55,113$ pixels, down-sampled by a factor of 4 in height and width. Patches extracted from the level-2 slide are used to emulate a pathologist, who typically does not use the full resolution when looking for cancerous regions. In this research, each patch had a size of $3 \times 224 \times 224$.

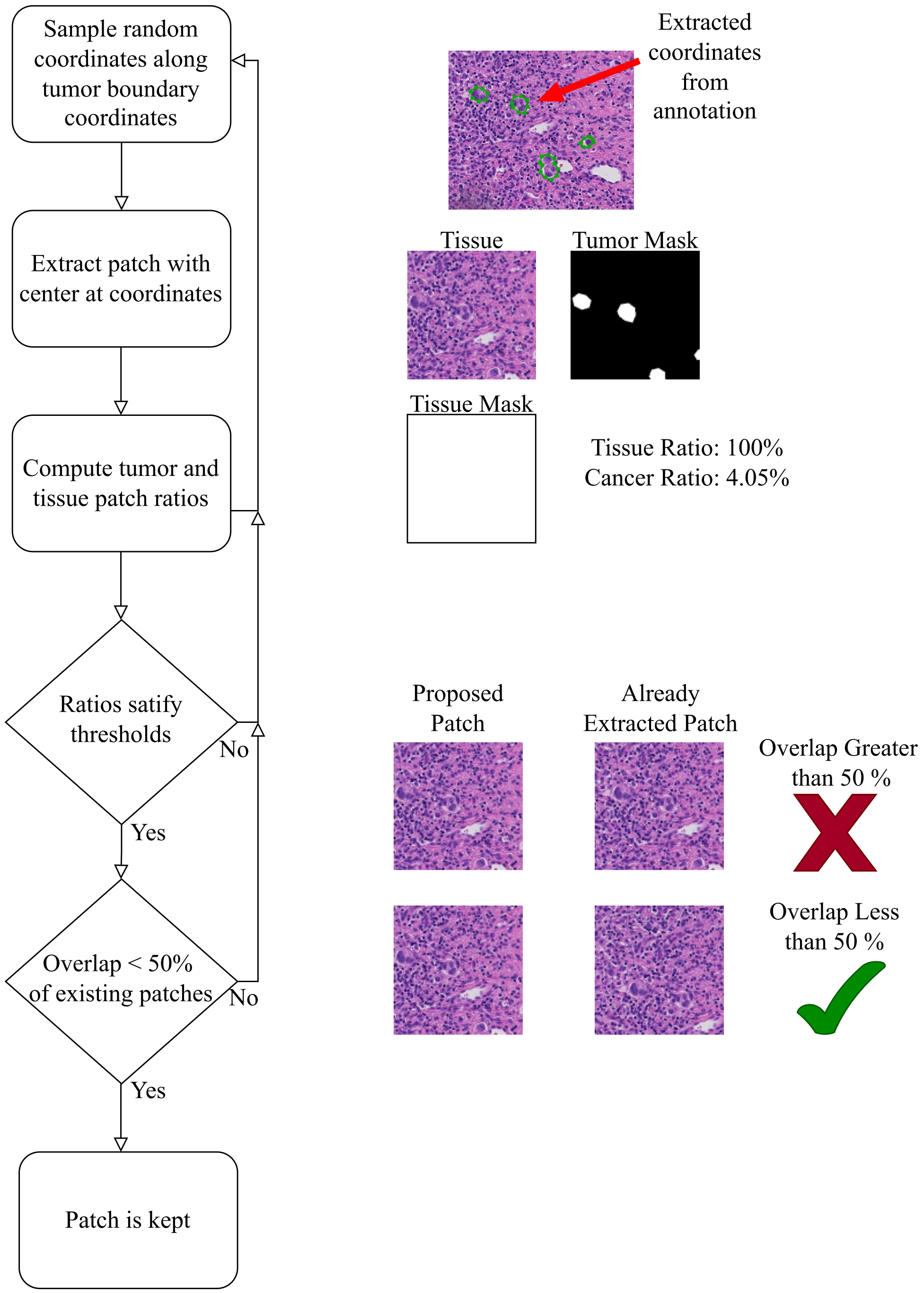

Supplementary Figure 2. A high-level diagram of the overall patch extraction process for tumor boundary patches.

Supplementary Table 1. The distribution of different patch types across the dataset splits. For non-cancer, cancer means a non-cancer patch that came from a slide with a cancer region while non-cancer means a non-cancer patch that came from a slide without a cancerous region.

<table>
<tr><td rowspan="4">Training
15,834</td><td rowspan="2">cancer
8,384</td><td>boundary: 7,203</td></tr>
<tr><td>nonboundary: 1,181</td></tr>
<tr><td rowspan="2">non-cancer
7,450</td><td>non-cancer: 4,000</td></tr>
<tr><td>cancer: 3,450</td></tr>
<tr><td rowspan="4">Validation
5,305</td><td rowspan="2">cancer
2,755</td><td>boundary: 2,392</td></tr>
<tr><td>nonboundary: 363</td></tr>
<tr><td rowspan="2">non-cancer
2,550</td><td>non-cancer: 1,400</td></tr>
<tr><td>cancer: 1,150</td></tr>
<tr><td rowspan="4">Testing
5,180</td><td rowspan="2">cancer
2,780</td><td>boundary: 2,385</td></tr>
<tr><td>nonboundary: 395</td></tr>
<tr><td rowspan="2">non-cancer
2,400</td><td>non-cancer: 1,150</td></tr>
<tr><td>cancer: 1,250</td></tr>
</table>

Supplementary Table 2. Models with Regularization Techniques to Control Overfitting

Model Type	L1	L2	Dropout	LR Decoder	LR backbone	LR MCA
Single MiT-B1(15.14M)	6e-4	6e-3	NAN	3e-6	1.875e-5	N/A
Single MiT-B2	6e-4	6e-3	NAN	3e-6	1.875e-7	N/A
CGS-Net MiT-B1	6e-4	6e-3	NAN	3e-6	1.875e-6	3e-5
CGS-Net MiT-B2	7e-4	7e-3	NAN	3e-6	1.875e-6	3e-6
Single SwinV2 Tiny	6e-4	6e-3	12.5%	3e-6	1.875e-7	N/A
Single SwinV2 Small	6e-4	6e-3	12.5%	3e-6	1.875e-7	N/A
CGS-NET SwinV2 Tiny	8e-4	8e-3	15%	3e-7	1.875e-7	3e-6
CGS-NET SwinV2 Small	8e-4	8e-3	15%	3e-7	1.875e-7	3e-6

Specific values for model hyperparameters are shown in Table 2. All models utilized the same data augmentation pipeline. A range of augmentations were explored and the one selected yielded the best results. Adam was used for optimization and all models have the same learning rate scheduler. All models have a batch size of 64 except for the CGS-Net SwinV2 Small model which had a batch size of 32. This was required due to the model size.

The averages, along with the standard deviations for the multi-cross attention modules, are shown in Figure 3. Note how the final Q, K, and V weights all have similar averages along with standard deviations. Recall that the Q weights are for the context feature tensors and the K and V weights are applied to the detail feature tensor.

Note that there is a sample bias present in all histopathological images. For example, in breast cancer, a patient is evidenced to have cancer after a medical professional has performed screening, followed by diagnostic scans, and then finally a biopsy [3]. The biopsy is analyzed by a pathologist to confirm if there is cancer. Thus, only patients with suspicious screenings and diagnostic scans receive a biopsy. The histopathological images are not from people without a suspicious scan, which results in an irremovable bias for all datasets. Also, for clinical applications, the process to extract patches from the entire WSI and model predictions will need further work as complex training patch extraction would not be needed.

Supplementary Table 3. Weight analysis of Multi-Cross Attention modules in the final CGS-Net weights. The Queries were initialized to 0 while the Keys and Values were initialized to 1. Average and standard deviations are provided.

Cross	Queries	Keys	Values
Weight Init	0	1	1
First Cross	$3.43e{-}09 \pm 9.41e{-}07$	$-1.42e{-}08 \pm 1.08e{-}06$	$2.59e{-}09 \pm 9.47e{-}07$
Second Cross	$7.88e{-}09 \pm 9.38e{-}07$	$-2.33e{-}09 \pm 1.11e{-}06$	$9.13e{-}09 \pm 9.51e{-}07$
Third Cross	$-4.84e{-}10 \pm 9.24e{-}07$	$-3.63e{-}09 \pm 9.06e{-}07$	$-2.97e{-}09 \pm 9.57e{-}07$
Fourth Cross	$1.85e{-}09 \pm 1.63e{-}06$	$-1.41e{-}09 \pm 1.18e{-}06$	$-8.12e{-}10 \pm 1.63e{-}06$

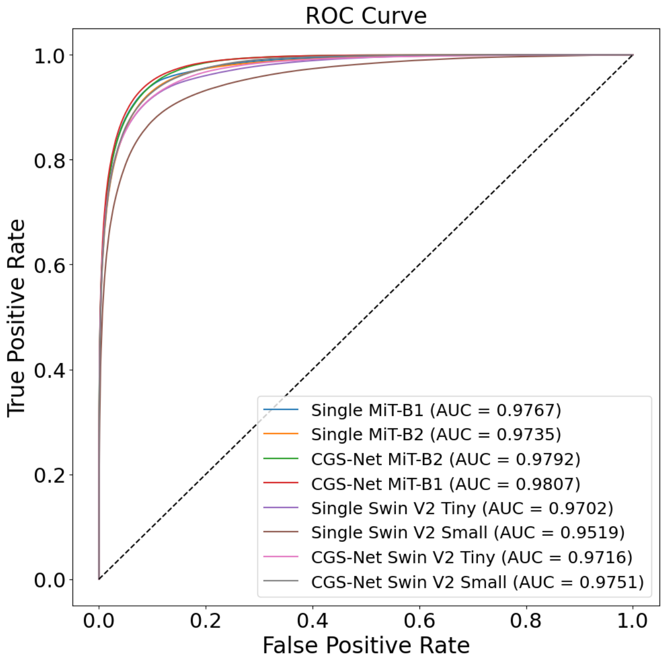

Supplementary Figure 3. The ROC curves for the models. Note that the CGS-Net architectures outperform their single-input model counterparts.

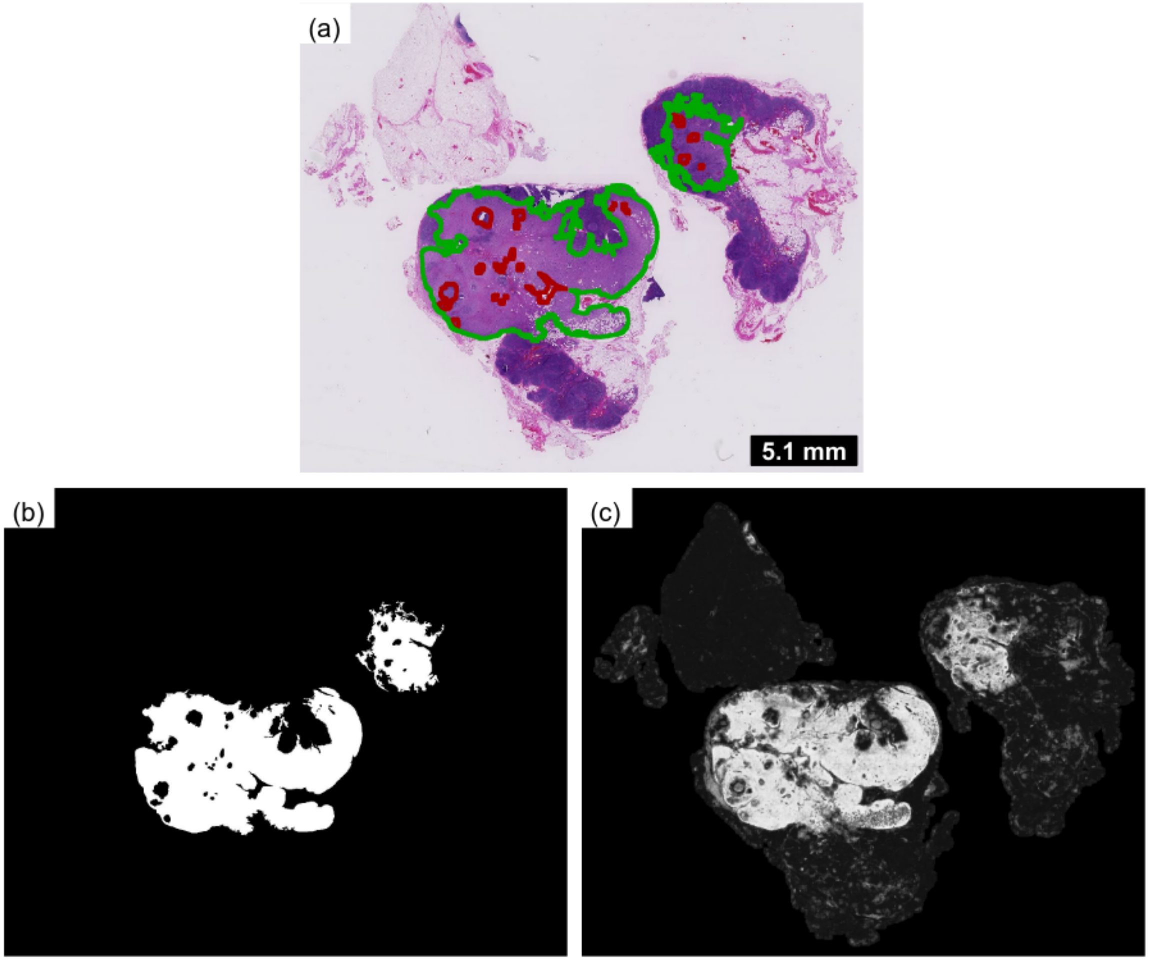

Supplementary Figure 4. (a) A WSI slide with a pathologist's annotation of the cancer regions. The green markings represent metastases with the red marking within denoting non-cancer areas. (b) A binary mask of the cancer tissue. This is the ground truth mask for the given WSI. (c) The prediction mask from CGS-Net Mit-B1. This involves extracting overlapping patches of size $3 \times 224 \times 224$ from the level-2 resolution and extracting the corresponding level-3 patches. Inputting both inputs into the model, and stitching together the model predictions to generate the entire slide prediction. The pixel intensities correspond to the pixel-level probabilities of being cancer.
